# Depression and survival of breast cancer patients: A protocol for systematic review and meta-analysis

**DOI:** 10.1097/MD.0000000000023399

**Published:** 2020-11-25

**Authors:** Guanghui Zhu, Juan Li, Jie Li, Xinmiao Wang, Minghao Dai, Jiayang Chen

**Affiliations:** a Oncology Department, Guang’anmen Hospital, China Academy of Chinese Medical Sciences; b Graduate School, Beijing University of Chinese Medicine; c Peking University Medical Science Center; d Peking Union Medical College, Beijing, China.

**Keywords:** breast cancer, depression, protocol, survival, systematic review

## Abstract

**Background:**

Breast cancer is the most common malignancy in women worldwide. Compared with other malignant tumors, breast cancer patients have a higher incidence of depression and other psychiatric symptoms. The purpose of this meta-analysis was to determine the association between long-term survival and depression in patients with breast cancer.

**Methods:**

This review will include cohort studies only. Multiple databases will be searched by 2 independent reviewers, including PubMed, EMBASE, the Cochrane Library, and PsycINFO. The language of studies should be English and Chinese, published from inception to the September 2020. Two independent reviewers will carry out literature screening, research selection and data extraction. Revman5.3 software will be used to generate funnel map, assess heterogeneity, make the subgroup analysis and complete sensitivity analysis.

**Results:**

This review will summarize the available evidence to determine the association between depression and survival in breast cancer patients.

**Conclusion:**

The results of this study will provide reference for the development of comprehensive treatment for breast cancer, and will promote further research.

**PROSPERO registration number:**

CRD42020202200

## 1 Introduction

Breast cancer is the most common malignant tumor in women all over the world. According to the 2018 global cancer epidemic statistics, there are about 2.089 million new cases of female breast cancer every year, and about 627,000 deaths from breast cancer in the same period.^[1]^ Even with advances in early screening and treatment, the death rate from breast cancer remains high.^[2–4]^ In order to prolong the survival time and reduce the mortality of breast cancer patients, it is important to identify the risk factors affecting the survival of breast cancer patients. A study from Singapore showed that depression significantly shortened the survival time of cancer patients and could predict the prognosis. Depression was associated with an increased mortality rate among breast cancer survivors in women (Hazard Ratio [HR]: 11.6, 95% confidence interval [95% CI]: 0.69–194.1, *P* = .089). The risk of depression-related death was very high among cancer patients who lived over 5 years after cancer diagnosis (adjusted HR: 4.69, 95% CI: 1.76–12.5, *P* = .002).^[5]^ After breast cancer diagnosed, the patients are worried about shortened survival time, recurrence and metastasis. Because most of them are female patients, they need to face impaired body image and decreased quality of sexual life caused by surgery and other treatments. Therefore, compared with other malignant tumors, the incidence of depression and other psychiatric symptoms in breast cancer patients are higher,^[6–8]^ which is also closely related to the lack of female secondary characteristics and physical symptoms such as nausea, vomiting, fatigue, hair loss, and insomnia caused by chemotherapy.^[9–13]^ Although the correlation between psychological factors and the survival of breast cancer patients has been extensively studied, the findings were not consistent. Some studies believed that depression was related to the shortened survival and increased mortality of breast cancer patients,^[14,15]^ while some studies believed that there was no correlation between the 2.^[16,17]^ A meta-analysis incorporating literature data found that depression was associated with a 30% increased risk of all-cause death in breast cancer patients, a 29% increased risk of specific death, and a 24% increased risk of breast cancer recurrence.^[18]^ However, the association between depression and breast cancer survival was not integrated.

Based on the above background, we designed this meta-analysis to collect and was to integrate data on survival, mortality, and risk of death in breast cancer patients with depression, in order to determine the correlation between long-term survival in patients with breast cancer and depression.

## 2 Method

This meta-analysis has been registered on PROSPERO (www.crd.york.ac.uk /prospero/) with number CRD42020202200, and referred to the guidance on conducting systematic reviews and meta-analyses of observational studies of a etiology.^[19]^ It will follow the reporting guidelines and criteria set in the Preferred Reporting Items for Systematic Reviews and Meta-analyses^[20]^ statement checklist and Meta-analysis Of Observational Studies in Epidemiology ^[21]^ checklist. Methods followed guidelines by the Cochrane Collaboration for the conduction of systematic reviews.^[22]^ Ethical approval is unnecessary for this study.

### 2.1 Inclusion criteria for study selection

#### 2.1.1 Study types

This review will include cohort studies only, including the vertical and horizontal designed with both prospective and retrospective studies.

#### 2.1.2 Participant types

The review will include patients with breast cancer who have been clearly diagnosed by imaging or pathology. Among them, patients diagnosed with depression should have the clear diagnosis time and methods. There will be no restrictions on age, gender, race, clinical stage, pathological type, and so on.

#### 2.1.3 Outcomes

(1) The median survival time (MST) of depressed and non-depressed breast cancer patients.
(2) The mortality rate of depressed and non-depressed breast cancer patients.
(3) The HR of death between depressed breast cancer patients and nondepressed ones.

#### 2.1.4 Exclusion criteria

Repetitive reports, conference summaries, case reports, review papers, or animal studies, and so on.

#### 2.1.5 Search strategy

Multiple databases will be searched by 2 independent reviewers, including PubMed, EMBASE, the Cochrane Library, and PsycINFO. The search terms include Breast Neoplasm, Breast Cancer, Breast Tumor, Mammary Cancer, Depression, Depressive, Mortality, Death, Survival, Case Fatality Rate, and so on. The language of studies should be English, published from inception to the September 2020. At the same time, reference lists of previous studies and reviews will be used as supplementary sources. PubMed database is taken as an example, and the retrieval strategy is shown in Table 1.

**Table 1:** Search strategy for the PubMed.

No.	Search terms
#1	((((((((((((((((“Breast Neoplasms”[Mesh]) OR (“Breast Neoplasm^∗^”[Title/Abstract])) OR (“Breast Tumor^∗^”[Title/Abstract])) OR (“Breast Cancer”[Title/Abstract])) OR (“Cancer, Breast”[Title/Abstract])) OR (“Mammary Cancer^∗^”[Title/Abstract])) OR (“Malignant Neoplasm of Breast”[Title/Abstract])) OR (“Breast Malignant Neoplasm^∗^”[Title/Abstract])) OR (“Malignant Tumor of Breast”[Title/Abstract])) OR (“Breast Malignant Tumor^∗^”[Title/Abstract])) OR (“Cancer of Breast”[Title/Abstract])) OR (“Cancer of the Breast”[Title/Abstract])) OR (“Mammary Carcinoma, Human”[Title/Abstract])) OR (“Human Mammary Carcinoma^∗^”[Title/Abstract])) OR (“Human Mammary Neoplasm^∗^”[Title/Abstract])) OR (“Breast Carcinoma^∗^”[Title/Abstract]))
#2	(((“Depression”[Mesh]) OR (“Depression^∗^”[Title/Abstract])) OR (“Depressive”[Title/Abstract]))
#3	((((((“Survival”[Mesh]) OR (“Mortality”[Mesh])) OR (“Survival^∗^”[Title/Abstract])) OR (“Mortality^∗^”[Title/Abstract])) OR (“Death^∗^”[Title/Abstract])) OR (“Case Fatality Rate^∗^”[Title/Abstract]))
#4	#1 AND #2 AND #3

### 2.2 Data collection and analysis

#### 2.2.1 Research selections

**Figure 1:**
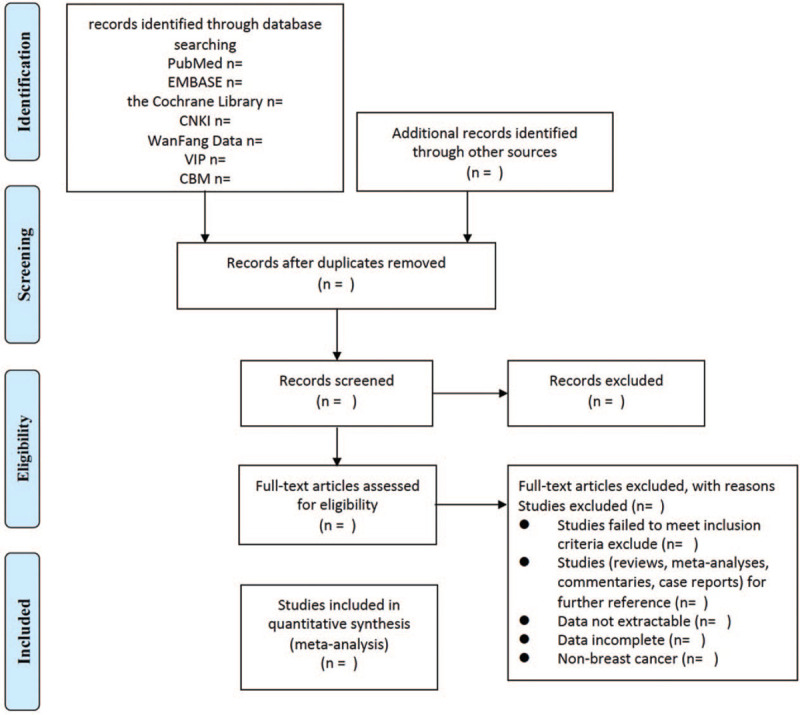
Flowchart of study selection.

Two independent reviewers will carry out literature screening, research selection and data extraction. The obtained documents will be imported into EndnoteX8, and the duplicate files will be searched and deleted first. Then, the title and abstract are screened, and studies that do not meet the inclusion criteria are deleted. After reading the full text of the remaining studies, the final included studies were determined. If the full text is not found, the corresponding author will be contacted. If there is a disagreement between the 2, the third reviewer will decide. The research selection process is shown in the Preferred Reporting Items for Systematic Reviews and Meta-analyses flowchart (Fig. 1).

#### 2.2.2 Extraction of data and information

The main data extracted by the 2 independent reviewers will include:

(1) The basic information included in the study, including the first author, publication time, area, published magazine, and impact factor.
(2) The baseline characteristics of the research object, including the sample size of each group, tumor stage, and diagnosis time and tools, follow-up time, and so on.
(3) Number of people diagnosed with depression, survivors, deaths, MST, the adjusted HR of death and 95% CI.

If the above data are not given directly in a study, the information in the study can be used to calculate. If the original data are not available or computable, this study will be excluded. A third senior author will help to reconcile any divergences between 2 authors.

#### 2.2.3 Heterogeneity assessment

Before the meta-analysis, the heterogeneity test was first performed using the *I*^2^ test. If the heterogeneity was small (*P* > .05 and *I*^2^ < 50%), the fixed effect model was used for analysis. The random effect model would be used, when the heterogeneity was large (*P* ≤ .05 or *I*^2^ ≥ 50%).^[23]^

#### 2.2.4 Evaluation of studying quality

Two researchers independently adopted the Newcastle–Ottawa scale^[24]^ to conduct a bias risk assessment on the included studies. Newcastle–Ottawa scale, with a total score of 9 stars, has a total of 8 items. If there was a disagreement, the 2 decided after a discussion; if it could not be resolved, a third researcher would assist.

#### 2.2.5 Assessment of reporting deviations

If necessary, we will examine the reporting bias using funnel plot and Egger regression test, when >10 trials are included.^[25,26]^

#### 2.2.6 Data synthesis

Pooled effect sizes were calculated for the outcome. To extract the MST in the included studies, we used the software STATA 15.1 software to calculate the natural logarithm of HR and the standard error. In each study, the number of depression and nondepressive breast cancer patients, the number of deaths and adjusted HR of death of depression breast cancer patients were extracted. Based on the above information, the natural logarithm of the correction effect value (Relative Risk [RR]/ HR) and standard error were calculated. RevMan 5.3 software was used for the meta-analysis, calculating the comprehensive effect value (RR/ HR) and 95% CI, to draw forest maps.

#### 2.2.7 Subgroup analysis

The subgroup analysis of the meta-analysis results for each outcome was required. The subgroup only includes items related to the study design, for example, stage, location, and follow-up time. The evaluation subgroup analysis will be conducted in accordance with the guidelines for credibility assessment.^[27]^

#### 2.2.8 Sensitivity analysis

The method of deleting studies one by one needed to be used to complete the sensitivity analysis of the results to ensure stability.

#### 2.2.9 Quality of evidence

The system grading of recommendations assessment, development, and evaluation was used in evaluating the evidence level of outcomes.^[28,29]^

## 3 Discussion

People is with the mental disorders, which predict unhealthy lifestyles such as smoking, alcohol consumption, insomnia, and so on, which may lead to cardiovascular disease and increase the risk of death.^[30]^ In addition, the treatment adherence of breast cancer patients diagnosed with depression is also one of the issues concerned by clinicians, such as difficulty in completing the full course of chemotherapy and endocrine therapy, leading to shortened survival time.^[31]^ Suicide is also one of the factors leading to shortened survival of breast cancer patients.^[32]^ Moreover, Kamiya A. et al found that chronic stress accelerated the growth and development of cancer by stimulating the sympathetic nerve in the tumor, affecting the survival of patients.^[33]^ Through this meta-analysis, we hope to integrate current data related to depression and survival of breast cancer patients, so as to provide reference for the development of comprehensive treatment for breast cancer.

## 4 Amendments

If amendments are needed, we will update our protocol to include any changes in the whole process of research.

## Author contributions

**Conceptualization:** Guanghui Zhu, Jie Li.

**Data curation:** Guanghui Zhu, Juan Li, Jie Li.

**Formal analysis:** Guanghui Zhu, Juan Li.

**Funding acquisition:** Jie Li.

**Investigation:** Guanghui Zhu, Jiayang Chen.

**Methodology:** Juan Li, Xinmiao Wang, Minghao Dai.

**Project administration:** Jie Li.

**Resources:** Guanghui Zhu, Juan Li.

**Software:** Xinmiao Wang, Jiayang Chen.

**Supervision:** Jie Li.

**Validation:** Guanghui Zhu, Minghao Dai.

**Writing – original draft:** Guanghui Zhu.

**Writing – review & editing:** Juan Li, Jie Li, Xinmiao Wang, Minghao Dai, Jiayang Chen.
